# Vickers micro-hardness study of the effect of fluoride mouthwash on two types of CAD/CAM ceramic materials erosion

**DOI:** 10.1186/s12903-022-02135-z

**Published:** 2022-03-30

**Authors:** Hamid Kermanshah, Elham Ahmadi, Niyousha Rafeie, Shiva Rafizadeh, Ladan Ranjbar Omrani

**Affiliations:** 1 Restorative Dentistry Department, Dental Research Center, Dentistry Research Institute, School of Dentistry, Tehran University of Medical Sciences, Tehran, Iran; 2 Dental Research Center, Dentistry Research Institute, School of Dentistry, Tehran University of Medical Sciences, Tehran, Iran; 3 School of Dentistry, Bushehr University of Medical Sciences, Bushehr, Iran; 4 Restorative Dentistry Department, School of Dentistry, Tehran University of Medical Sciences, North Kargar, Tehran, 14174 Iran

**Keywords:** Hardness, Computer-aided design, Gastric acid, Sodium fluoride, Ceramics

## Abstract

**Background:**

The aim of this study was to evaluate the protective effects of fluoride mouthwash on the surface micro-hardness of two types of CAD/CAM ceramics after exposure to acidic solutions.

**Methods:**

40 samples (5 × 5 × 3 mm^3^) were prepared from two different ceramics: Vitabloc Mark II CAD, and IPS e.max CAD. The samples were randomly divided into 5 groups in each ceramic (n = 8) immersed in different solutions: G_s_: saliva: G_GA_: gastric acid, G_AA_: acetic acid, G_FGA_: sodium fluoride + gastric acid, G_FAA_: sodium fluoride + acetic acid. The microhardness of samples was measured before and after immersion in different solutions by Vickers microhardness tester. By subtracting the microhardness values after and before immersion, the microhardness changes of the samples were obtained. Data were analyzed by Two-way analysis of variance, one-way analysis of variance, and Tukey test (α = 0.05).

**Results:**

Immersion in different solutions reduced the microhardness. Microhardness loss was significantly affected in G _FAA_ and G _FGA_ groups in both types of ceramics (*P* < 0.05). For Vitabloc Mark II groups, the microhardness loss was significantly higher in G_FAA_ and G_FGA_ compared to IPS e.max CAD *P* < 0.001).

**Conclusion:**

Fluoride mouthwash in conjunction with acidic solutions may adversely affect microhardness of Vitabloc Mark II CAD, and IPS e.max CAD that may consequently compromise the clinical service. Vitabloc Mark II CAD was significantly more affected than IPS e.max CAD.

**Supplementary Information:**

The online version contains supplementary material available at 10.1186/s12903-022-02135-z.

## Background

Due to changes in people's lifestyles, erosive lesions have become one of the most critical dental problems. Dental erosion is defined as irreversible loss of tooth tissue due to acidic agents with a non-bacterial origin. These acids have external (nutrition, medications) or internal (gastroesophageal reflux disease (GERD), bulimia, and nausea) origin [1].

Early signs of erosion include smooth facets at the facial, palatal, and occlusal surfaces. Further progression of lesions results in hypersensitivity, esthetic problems, and loss of vertical dimension [1]. In these cases, for proper treatment, the causative agents must be identified and controlled; one of the controlling factors of erosion is fluoride compounds which cause fluoride deposits on tooth surfaces and prevent further dissolution. Sodium fluoride (NaF) protective effect against dental erosion may be due to physically protecting calcium fluoride barrier deposition on the tooth surface. This barrier act as a sacrificial coating in the acid events [2, 3]. In addition to tooth structures, fluoride might also affect dental materials including dental ceramics; Filho et al. reported that the microhardness of lithium disilicate ceramic samples decreased after immersion in NaF solution. It has been speculated that NaF mouthwash is able to cause dissolution of the glass phase in dental ceramics. NaF can be dissociated into sodium (Na^+^) and fluoride (F^−^) ions in aqueous environments. Sodium ions weaken the strong, linear bonds of Si–O-Si and make the bond more susceptible to be broken by F^−^ ions which eventually results in the formation of tetrafluorosilane (SiF4) [4].

Among different dental materials used for restoring esthetic and function in cases of extensive teeth erosion, dental ceramics such as IPS e.max CAD and Vitabloc Mark II CAD are a choice for repairing anterior and posterior teeth due to their tissue compatibility, aesthetics, and high abrasion resistance [5]. Moreover, in the last three decades, use of CAD/CAM (computer-aided design and computer-aided manufacturing) technology has increased significantly due to its advantages including saving time, reducing possible errors during construction, and increasing accuracy [6, 7] all of which have resulted in increased use CAD/CAM restorations in dental practice.

According to the literature, IPS e.max CAD Ceramic (lithium disilicate glass–ceramic) has high strength and good edge integrity and is popular due to the possibility of creating appearance characteristics by using post-machining painting [8]. Vitabloc Mark II CAD (feldspar ceramics) is mainly composed of silicon dioxide (silica or quartz) with varying amounts of alumina. Due to the small particle size (average 4 micron) and sintering process. It is reported that this type of ceramic has the highest abrasion resistance [9].

Microhardness is a material's surface properties, defined as the resistance of a material to indentation, scratching, or permanent surface penetration. Microhardness is usually related to materials' mechanical properties and is an essential property of dental materials associated with compressive strength and resistance to softening [10]. Low cost and being easy application may cause this method to be popular in evaluating dental erosion [11].

Despite the advantages of ceramic restorations, previous studies have reported that chemical stability and surfaces of ceramic restorations can be negatively affected by oral cavity environment and erosive agents [4, 12]. Organic acids such as acetic acid and citric acid may have fairly corrosive potential due to their chelating effect on ceramics [13]. In a previous study conducted by Farhadi et al. [14] it was reported that the surface roughness of two types of CAD/CAM ceramics increased after exposure to acidic environment followed by immersion in sodium fluoride solution. Kukiattrakoon et al. [15] have concluded that acidic agents used in their study decreased the surface roughness of four types of tested ceramics. On the other hand, the behavior of ceramic in contact with gastric acid in studies is inconsistent with different exposure times and study design [14, 16].

Many previous studies [15, 17, 18] have evaluated effect of acidic solutions on the surface roughness and microhardness of dental ceramic; however, to the best of our knowledge, no study has investigated the effect of sodium fluoride mouthwash on the microhardness of dental ceramics subjected to erosion.

Microhardness evaluations are performed using indentation tests (with Vickers or Knoop indenters) that show the material resistance to localized plastic deformation [17]. Both Vickers and knoop tests employ loads less than 9.8 N. The resulting indentations are small and limited to depths of less than 19 µm. As results, these tests are suitable for measuring the hardness in small samples [19].

Thus, due to the protective effect proposed for fluoride and the different behavior of ceramics in varied acidic environments, the aim of this study was to evaluate the effect of sodium fluoride mouth wash on microhardness of two types of CAD/CAM ceramic materials after immersion in acidic solutions. The null hypothesis was that NaF mouth wash has no effect on the microhardness of Vita mark II CAD and IPS e.max CAD after immersion in acidic solutions.

## Methods and materials

The microhardness of two types of CAD/CAM ceramics was evaluated in this experimental study: Vita mark II CAD (Vita Zahnfabrik, BadSackingen, Germany) and IPS e.max CAD (Ivoclar Vivadent, Liechtenstein, Zurich, Switzerland). Table 1 summarizes the characteristics of the materials used in this study. According to Kukiattrakoon et al. study [13], using one-way ANOVA Analysis option in PASS software for microhardness variable, considering α = 0.05, β = 0.2, mean standard deviation equal to 0.48, and effect size equal to 0.45, the minimum sample size for each group was considered to be 8.

**Table 1:** Materials characteristics used in the study

Material	Ceramim type	Composition	Manufacturer
IPS e.max CAD	Lithium disilicate ceramic	57–80% SiO_2_, 11–19% Li_2_O, 0–13% K_2_O, 0–11% P_2_O_5_, 0–8% ZrO_2_, 0–8% ZnO, 0–5% Al_2_O_3_, 0–5% MgO	Ivoclar
VITABLOC MARK II	Feldspathic ceramic	56–64% SiO_2_, 20–23% Al_2_O_3_, 6–9% Na_2_O, 6–8% K_2_O, 0.3–0.6% CaO. TiO_2_ < 0.1	Vita Zahnfabrik, Bad Säckingen, Germany

From each type of ceramics, 40 samples (5 × 5 × 3 mm) were prepared from ceramic blocks using a diamond-coated cutting disc (Mecatome T201A, Presi, Grenoble, France) with water coolant. The samples' dimension accuracy was evaluated with an electron caliper (Mitutoyo Co., Kanagawa, Japan) with an accuracy of 0.1 mm.

IPS e.max CAD samples were crystallized using an electric oven (Programator P300, Ivoclar Vivadent, Liechtenstein) at 850° C for 30 min.

One side of all samples was polished using silicon carbide abrasives 600, 400, 800, 1000, 1200, and 1500 grit, in a polishing machine (Malek Teb, Iran) under sufficient water flow.

Next, the microhardness value was measured by a Vickers microhardness tester (Micromet II, Buehler LTD., Lake Bluff, IL. USA) equipped with a Vickers diamond. First, three indentations were placed by a diamond indenter applying 200 g load for 15 s. These indentations were placed 500 µm apart from each other [18]. Then, an optical microscope (under 40 × magnification) measured each indentation's two diagonal lengths to calculate the microhardness. The microhardness was calculated using the following formula [20], and the average of the three measurements was calculated and reported as the microhardness value in Vickers hardness (VHN) units.

$$Hv = \frac{1.8544 \times P}{{d^{2} }}$$

Hv is the Vickers hardness number in kg/mm^2^, P is the indenter load in kg and d is the diagonal length of the impression in mm.

After measuring the microhardness values, ceramic blocks were randomly divided into five groups and each sample was immersed in sealed tube containing 20 ml of solution according to its groups:

G_S_ (saliva group/negative control): In this group, the samples were immersed in artificial saliva and incubated at 37 °C for 168 h. The used artificial saliva was prepared by 1 M sodium chloride (NaCl), 0.2 M monosodium phosphate (NaH_2_PO_4_), 1 M acetic acid, 0.2 M calcium chloride (CaCl_2_), and 2.0% sodium azide (NaN_3_) at pH = 6.3 [14].

G _GA_ (gastric acid group): In this group, the samples were immersed in reconstituted gastric acid solution (HCl) and incubated at 37 °C for 168 h (equal to 22 years of ceramic service in a patient with GERD [21]). For this purpose, simulated gastric acid (HCl) was made by dissolving 2.0 g of sodium chloride and 3.2 g of pepsin in 7.0 ml of hydrochloric acid and water until it reached a total volume of 1000 ml. The pH of the resulting solution was equal to 1.14.

G_AA_ (acetic acid group): the samples were immersed in acetic acid for 168 h in an oven at 80 °C. The acetic acid was 4% and was prepared by diluting 100% acetic acid to pH 2.45.

G_FGA_ (fluoride + gastric acid group): In this group, first, the samples were immersed in Fluorigard mouth wash (Colgate-Palmolive) containing 0.05% sodium fluoride (225 ppm fluoride) with PH = 5.9 and then, the samples were incubated at 37 °C for 69 h (equivalent to daily rinsing with a solution for 30 s for 22 years). Afterward, the samples were immersed in HCL and incubated at 37 °C for 168 h.

G_FAA_ (fluoride + acetic acid): The samples were immersed in Fluorigard and incubated at 37 °C for 69 h followed by immersion in acetic acid and for 168 h in an oven at 80 °C.

**Fig. 1:**
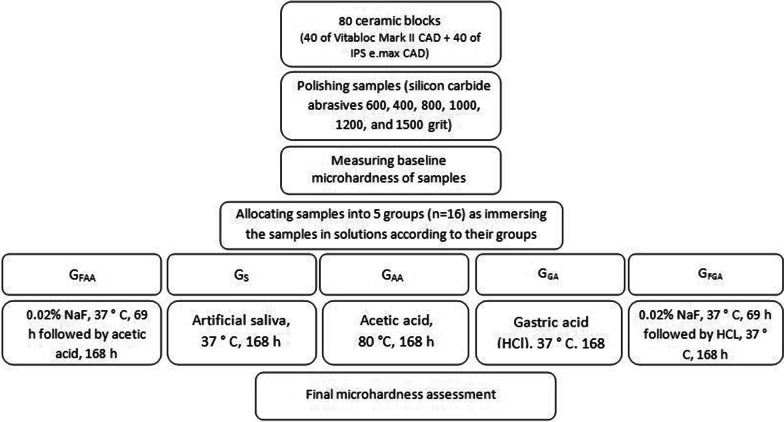
Methodology used in the present study

After the storage period, the samples were entirely rinsed with water for 20 s. Next, the microhardness value was measured for each sample. By subtracting the microhardness values after and before immersion, the microhardness changes of the samples were obtained. Figure 1 summarizes the methodology used in this study.

Data were analyzed by SPSS statistical software for social sequences version 0.22. Two-way analysis of variance was used to measure the effects of storage environment type, ceramic type, and microhardness interactions. A comparison of microhardness changes in different environments in two types was performed by one-way analysis of variance, and comparisons between groups were conducted by Tukey test. The first type's error rate in the present study was equal to 0.05 (α = 0.05).

## Results

There were significant differences in terms of the microhardness loss values of different groups of Vitabloc mark II CAD (*p* < 0.0001) and IPS e-max CAD (*p* < 0.0001).

The mean surface hardness loss values of Vitabloc mark II and IPS e-max ceramic are presented in Table 2. In Vitabloc mark II, the highest microhardness reductions were observed in G_FAA_, followed by G_FGA_, G_AA_, G_GA_, and G_S_. In e-max ceramic, the highest microhardness reductions were observed in G _FGA_, followed by G_FAA_, G_AA_, G_GA_, and G_S_.

**Table 2:** Mean and standard deviation of microhardness values before and after immersion in different solutions

Groups	VITABLOC MARK II		IPS e.max CAD	
	Before	After	Before	After
G_s_	747.59 ± 20.37	688.26 ± 190.46	733.78 ± 15.75	737.05 ± 18.44
G_GA_	689.79 ± 44.48	606.58 ± 106.09	718.41 ± 34.19	670.23 ± 24.6
G_AA_	716.56 ± 56.16	600.78 ± 100.76	713.70 ± 31.42	663.70 ± 49.26
G_FGA_	740.30 ± 30.63	316.00 ± 130.55	744.23 ± 18.94	598.46 ± 39.57
G_FAA_	739.01 ± 29.67	237.41 ± 23.83	725.90 ± 14.82	623.40 ± 82.86

G_S_, immersion in artificial saliva; G_GA_, immersion in gastric acid; G_AA_, immersion in acetic acid; G_FGA_, immersion in Fluorigard rinsing followed by HCL; G_FAA_, immersion in Fluorigard followed by acetic acid

Regarding the solution type, according to the results of student t-test, significant differences were observed in microhardness loss values of Vitabloc mark II and IPS e-max ceramic in G_FGA_ (*p* < 0.0001) and G_FAA_ (*p* < 0.0001) groups. Microhardness loss values in G_GA_ (*p* = 0.37), G_AA_ (*p* = 0.17) and G_S_ (*p* = 0.36) were not significantly different from each other.

**Fig. 2:**
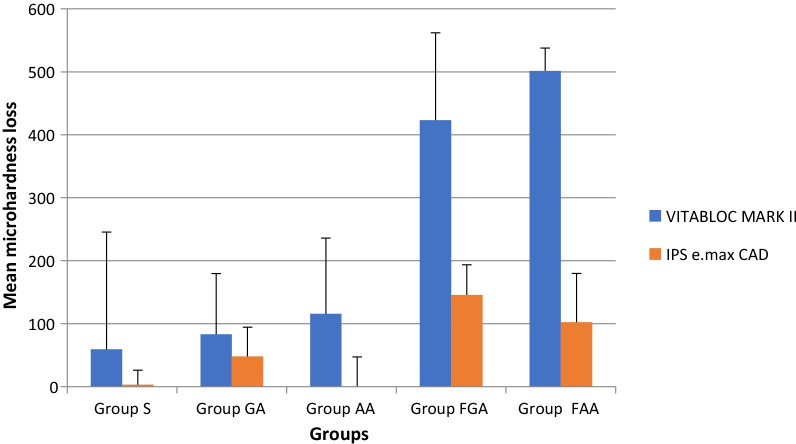
Mean and standard deviation of microhardness loss of tested ceramics after immersion in different mediums

Comparing two types of ceramics, according to independent samples t-test results, the loss of microhardness in G_FGA_ and G_FAA_ in Vitabloc mark II was significantly higher than e.max ceramic (*p* + 0.001) (Table 3). Figure 2 illustrates the mean and standard deviation of microhardness loss of the tested ceramics after immersion in different solutions.

**Table 3:** Mean and standard deviation of microhardness loss after immersion in different mediums

Groups	VITABLOC MARK II	IPS e.max CAD	*P* value
G_s_	− 59.32 ± 186.26^B,a^	3.27 ± 22.98^B,a^	0.36
G_GA_	− 83.21 ± 96.47^B,a^	− 48.18 ± 46.33^B,a^	0.37
G_AA_	− 115.78 ± 120.34^B,a^	− 50.00 ± 46.71^B,a^	0.17
G_FGA_	− 423.30 ± 138.73^A,a^	− 145.76 ± 47.73^A,b^	0.0001
G_FAA_	− 501.60 ± 36.17^A,a^	− 102.50 ± 77.32^A,b^	0.0001

G_S_, immersion in artificial saliva; G_GA_, immersion in gastric acid; G_AA_, immersion in acetic acid; G_FGA_, immersion in Fluorigard rinsing followed by HCL; G_FAA_, immersion in Fluorigard followed by acetic acid. In each column, the same uppercase letters are statistically similar. In each row the same lowercase letters are statistically similar

## Discussion

Complete oral reconstruction in patients with extensive erosive dental lesions is one of the complex treatments in dentistry. Unfortunately, many teeth are usually damaged in these patients, and their aesthetic and function are lost [22]. Ceramic materials are chosen due to low thermal conductivity, good biocompatibility, and low plaque accumulation [23]. However, ceramic materials are exposed to different temperatures and acidic changes associated with various acidic foods and beverages and gastroesophageal reflux disease (GERD) in the oral environment. Therefore, ceramics must show sufficient resistance [13]. Among different ceramics, feldspathic porcelain and IPS e.max ceramics create higher esthetic results [24], this is the reason why we chose these two ceramics for evaluation in our study.

The present study showed that following immersion of the samples in different solutions, their microhardness values reduced. In Vitabloc Mark II CAD and e.max CAD, the highest reduction in microhardness occurred in G _FGA_ and G _FAA_ groups, which were significantly higher than in other groups, and thus, the null hypothesis was rejected.

The acetic acid and simulated gastric acid decreased the microhardness of both types of ceramics. However, the reduction did not significantly differ from GS; this finding was in line with Cruz et al.'s study. They simulated about two years of ceramics exposure in a patient with GERD. They suggested that a longer exposure time might have a significant effect on ceramics [25]. We used a longer exposure time to simulate about 22 years of exposure in a patient with GERD due to the service time for dental ceramics [21]. Kikiattrakoon et al. showed that after immersion of different ceramics in gastric acidic solutions, their surface hardness experienced significant reductions [13]. However, it seems that CAD/CAM ceramics have smaller and more homogenous crystal sizes with increased interlocking between them, and smoother boundaries to surrounding glass phase which makes them more resistant to acid dissolution in comparison to conventional versions [26, 27].

In our study, the microhardness values of both types of CAD/ CAM ceramic decreased after immersion in acetic acid and gastric acid. These findings are in line with those of Colombo et al. who concluded that immersion in Coca-Cola for 7 days negatively affected the microhardness of CAD/ CAM ceramics [17]. It has been suggested that a decrease in microhardness values of ceramic following immersion in acid is due to the dissolution of ceramic [28]; In an aqueous solution, hydrogen or hydronium ions (H_3_O+) diffuse from the solution into the glassy matrix of ceramic and conversely, alkali ions diffuse from glass matrix to aqueous solution. It is probable that some channels or pores form in glass matrix due to the diffusion of these alkaline ions. These pores and channels might increase the diffusion of water molecules and develop areas showing localized breakage in Si–O–Si bond. Following breakage in Si–O–Si bonds, silicon is released from glassy matrix that results in impairment of the entire ceramic structure [15].

Previous studies have evaluated the effect of fluoride on different restorative materials; Yu et al. [29] investigated the effect of topical fluoride application on different restorative materials and reported that the acid resistance of poly acid modified resin composite and glass ionomer cement increased after application of amine fluoride (AmF) and stannous fluoride (SnF_2_). They concluded that the observed phenomenon was due to the formation of a fluoride-rich layer on the surface of poly acid modified resin composite and glass ionomer cement. In another study, it was shown that the microhardness of IPS e.max ceramic decreased after immersion in NaF and acidic solution. These findings are in line with those of the present study. In our study, the microhardness values of both types of ceramics decreased significantly after immersion in acid and NaF. It is believed that sodium fluoride might dissociate to sodium (Na^+)^ and fluoride (F^−^) ions in aqueous solutions. In turn, sodium ions weaken the bond between silicon and oxygen in silicon dioxide and make it more susceptible to bond breakage by fluoride ions which lead to the formation of tetrafluorosilane (SiF_4_) [4]. Since the glass phase of ceramics is mostly composed of silicon dioxide, the breakage of Si–O–Si bond and formation of SiF4 in combination with the dissolution of ceramic glassy phase would be responsible for significantly decreased microhardness values after immersion in acid and NaF solution.

Additionally, fluoride concentration in mouthwash composition might affect its potential efficacy against erosion. Carey et al. [30] reported that 25 ppm was optimal fluoride concentration capable of protecting dentin from a 1.00% citric acid challenge. We used a fluoride mouthwash containing 0.05% sodium fluoride (225 ppm fluoride). However, there is no evidence suggesting the optimal fluoride concentration in order to protect ceramics from further erosion caused by acidic solutions. It is possible that the fluoride concentration we used was not sufficient to protect the ceramic erosion. PH of the mouthwash is another determining factor which might affect dentin, enamel and restorative materials; it was reported that mouthwashes with a pH lower than the critical pH of enamel and dentin present an erosive potential on dentin [31]. However, evidence regarding the effect of mouthwash PH on the erosion and hardness of restorative materials is still scarce.

In this study, the microhardness values of Vitabloc Mark II decreased after immersion in artificial saliva while the microhardness of IPS e.max slightly increased. However, the microhardness change in both ceramics was not statistically significant. It seems that the effect of saliva storage on the microhardness values depends on the ceramic composition; a recent study showed that surface microhardness of Lava Ultimate decreased significantly by increasing its storage duration in water. In contrast, no change was observed in microhardness of Vitablocs Mark II irrespective of storage duration [32]. In another study conducted by Fahmy et al. [33], the surface microhardness of a hydrothermal low-fusing glass–ceramic (Duceram LFC) increased after 3 weeks of storage in artificial saliva. It is possible that the increase in microhardness of IPS e.max is related to its different composition from Vitabloc Mark II.

Regarding the ceramic type, the microhardness loss in G _FAA_ and G _FGA_ groups in Vitabloc Mark II ceramics was significantly higher than e.max CAD. It seems that crystal-based ceramics such as lithium disilicate are more resistant in fluoride solutions than glass-based ceramics (feldspathic). IPS e.max CAD is a lithium disilicate glass in terms of microstructure, and it contains a glass matrix with 70% crystals in the size of 1.5 microns, including lithium metasilicate, disilicate, and phosphate crystals [34, 35]. Its Lithium-containing glass is a tri-phase Li–Si–K–O with zirconium oxide that is more resistant to acid-induced corrosion [27, 36]. On the other hand, VITA BLOC Mark II CAD has a feldspar structure with irregular crystal phases of 4 microns in size. These crystalline phases include insoluble feldspars, lucite crystals, and alumina particles placed in a weak glassy feldspar matrix. Due to its heterogeneous microstructure, the surface of this ceramic is non-uniformly damaged which leads to a reduction in its microhardness [33, 34].

According to a previous study [14], exposure to acidic solutions and sodium fluoride mouth wash significantly increased the surface roughness parameters of Vitablocs Mark II and IPS e.max CAD compared to immersion in saliva. These findings were also confirmed by SEM evaluations. According to our result, surface microhardness values of Vitablocs Mark II and IPS e.max CAD were negatively affected by the exposure to an acidic environment and sodium fluoride mouth wash. Regarding the results of the present study and those reported by the previous study [14], preventive measures involving the fluoride should be used in patients with bulimia or GERD more cautiously.

This should be noted that the present study is an in vitro study that might not simulate the actual complexity of the oral environment affected by the oral cavity's pH, saliva composition, and dilution and buffering effects of saliva on acidic drinks and foods, temperature, patient's age, and gender. Moreover, the solutions in which the samples were immersed were static environments with acidic to neutral pH unlike the oral cavity conditions. The cyclical immersion of the samples would be beneficial in order to mimic the intraoral condition more accurately. In addition, artificial saliva used in the present study lacks salivary proteins which might affect the microhardness of ceramics in the oral environment and thus, generalization of the results obtained from this study to the clinical setting must be done with caution. In addition, we measured the PH of the solutions at the beginning of the study; however, evaluating PH through the course of the study would be recommended in the future studies. Future studies simulating the oral cavity environment using thermocycling procedure would be also advantageous to confirm the results of the present study.

## Conclusion

Within the limitations of this in vitro study, it can be concluded that fluoride mouthwash in conjunction with acidic solutions might decrease the microhardness of Vitabloc Mark II CAD and IPS e.max CAD which should be taken into account when patients suffering from GERD or other erosive conditions require ceramic restorations.

## Supplementary Information

**Additional file 1.** Raw data and analysis.

## Data Availability

All data generated or analysed during this study are included in this published article and Additional file 1.
